# Situation analysis of onchocerciasis in Cameroon: a protocol for systematic review of epidemiological studies and impact of disease control interventions

**DOI:** 10.1186/s13643-020-1287-y

**Published:** 2020-02-11

**Authors:** Hugues C. Nana-Djeunga, André Domche, Yannick Niamsi-Emalio, Henri C. Moungui, Martin Walker, Maria-Gloria Basáñez, Joseph Kamgno

**Affiliations:** 1 Centre for Research on Filariasis and other Tropical Diseases (CRFilMT), Yaoundé, Cameroon; 2 Parasitology and Ecology Laboratory, Department of Animal Biology and Physiology, Faculty of Science, University of Yaoundé I, Yaoundé, Cameroon; 3 National Onchocerciasis Control Program (NOCP), Yaoundé, Cameroon; 4 Department of Pathobiology and Population Sciences and London Centre for Neglected Tropical Disease Research, Royal Veterinary College, Hatfield, London UK; 5 Department of Infectious Disease Epidemiology, London Centre for Neglected Tropical Disease Research and MRC Centre for Global Infectious Disease Analysis, Imperial College London, London, UK; 6 Faculty of Medicine and Biomedical Sciences, University of Yaoundé I, Yaoundé, Cameroon

**Keywords:** Onchocerciasis, Elimination, Cameroon, Systematic review, Protocol

## Abstract

**Background:**

Many control methods have been implemented to tackle onchocerciasis and great successes have been achieved, leading to a paradigm shift from control of morbidity to interruption of transmission and ultimately elimination. The mandate of the African Programme for Onchocerciasis Control (APOC) ended in 2015, and endemic countries are to plan and conduct elimination activities by themselves, with technical assistance by the Expanded Special Project for Elimination of Neglected Tropical Diseases (ESPEN). To this end, an elimination expert committee was set up in Cameroon in 2018. This committee identified the need to update the data on the current situation of onchocerciasis. The present study aims to systematically review and report all available epidemiological data, including prevalence, intensity and transmission of onchocerciasis to provide pertinent information that will be useful to design optimal strategies to achieve onchocerciasis elimination in Cameroon.

**Methods:**

PubMed/MEDLINE, EMBASE and Web of Science will be searched from inception onwards. Grey literature will be identified through Google Scholar searches, dissertation databases and other relevant documents such as government reports. Eligible studies will be mostly observational, including cohort and cross-sectional surveys. No limitations will be imposed on publication status and study period. The primary outcomes will be (1) the prevalence and intensity of *Onchocerca volvulus* infection in humans, (2) transmission intensity and (3) impact of interventions on prevalence, intensity and transmission of onchocerciasis. Secondary outcomes will be environmental and socio-demographic factors supporting the primary outcomes. Two reviewers will independently screen all citations, full-text articles and abstract data. Potential conflicts will be resolved through discussion. Methodological quality including bias will be appraised using appropriate approaches. A narrative synthesis will describe quality and content of the epidemiological evidence. Prevalence and intensity of infection estimates will be stratified according to gender, age, geographical location and year of publication.

**Discussion:**

This study will provide the health authorities as well as the scientific community with up-to-date information about the epidemiological situation of onchocerciasis in Cameroon. Understanding the spatiotemporal dynamics of the infection will help to define alternative and complementary strategies to accelerate onchocerciasis elimination in the country. Results of this review will also be used to update existing epidemiological models for onchocerciasis in order to fine-tune predictions of elimination timeframes in the country.

**Systematic review registration:**

This protocol is under registration review in PROSPERO.

## Introduction

### Rationale

The paradigm of the fight against onchocerciasis has shifted from morbidity control to interruption of transmission and ultimately elimination since 2010 in Africa [1], motivated by the demonstration in some foci that onchocerciasis can be eliminated by long-term annual (or biannual) mass drug administration (MDA) of ivermectin [2–4]. Despite this enthusiastic and optimistic declaration and commitment by control programmes and stakeholders, the truth is that the infection still persists in many endemic foci in Africa [5, 6]. A previous study documenting epidemiological trends mainly in the countries of the former Onchocerciasis Control Programme in West Africa (OCP) area indicated that ivermectin alone would not help achieving onchocerciasis elimination in some foci before 2030 [7]. Mathematical modelling has also indicated that in the most highly endemic foci, ivermectin alone will not be sufficient to eliminate the infection [8, 9]. It is now unanimously accepted that alternative and complementary treatment strategies (ATS) will certainly be necessary in some epidemiological settings [10], and Boussinesq and colleagues have provided detailed information on the different options to be considered to accelerate onchocerciasis elimination [11]. Therefore, an essential step to be taken is the situation analysis of onchocerciasis in each endemic country to identify the appropriate suite of interventions that need to be implemented to achieve interruption of transmission, if not by 2020 (the year proposed by the World Health Organization (WHO) in its 2012 roadmap on neglected tropical diseases [12], by 2025 (when 80% of African endemic countries should achieve elimination as suggested by the Joint Action Forum of the WHO/African Programme for Onchocerciasis Control (APOC) (JAF 2012, Final Communiqué). Cameroon in particular is a country with great ecological and epidemiological diversity, not only for onchocerciasis but also for other filarial infections that make it difficult to implement ivermectin MDA. This study is a systematic review aiming to collate all existing data on onchocerciasis epidemiology in relation to the interventions that have been implemented and the environmental determinants of its transmission in Cameroon.

### Objectives

The main objective of this systematic review will be to report spatiotemporal trends in prevalence, intensity and transmission of *Onchocerca volvulus* infection in Cameroon. The secondary objective will consist in collating interventions and socio-demographic and environmental data in order to elucidate their impact on prevalence, intensity of infection and transmission of onchocerciasis in Cameroon.

The goal is to provide the NOCP and the national committee for elimination of onchocerciasis and lymphatic filariasis (NCEOLF) with robust and reliable data that can be analysed to make informed decisions on context-specific current or alternative treatment strategies that need to be implemented to achieve the WHO/APOC goal of onchocerciasis elimination by 2025 and beyond.

The study therefore intends to address the following questions:

1. What are the trends in prevalence, intensity of infection and transmission of onchocerciasis in Cameroon from baseline and following the inception of interventions onwards?
2. What is the impact of different control interventions on infection prevalence, intensity and transmission of onchocerciasis in Cameroon?
3. What are the potential factors underlying the trends in prevalence, intensity of infection and transmission of onchocerciasis in Cameroon?

## Methods

This review protocol was reported in accordance with reporting guidance provided by the Preferred Reporting Items for Systematic Reviews and Meta-Analyses Protocols (PRISMA-P) statement [13] (see PRISMA-P checklist in Additional file 1). In addition, the process of registering the review protocol with the PROSPERO database was initiated (registration reference number: 158962; registration process ongoing in PROSPERO).

### Eligibility criteria

No restrictions will be made on the time horizon of the studies. In contrast, any cohort or cross-sectional study, either published or unpublished, assessing the endemicity of *Onchocerca volvulus* infection (prevalence and/or intensity or transmission), as well as the interventions used to control this filarial disease in Cameroon, will be eligible for this systematic review. Studies involving either blackfly vector (*Simulium* spp.) or human population whatever their age and gender will be targeted. However, case reports, clinical trials, editorials, letters to the editor, systematic reviews or meta-analyses will be excluded. Search languages will be limited to French and English, the Cameroon official languages in which almost all publications are done.

### Information sources and search strategy

PubMed/MEDLINE, EMBASE and Web of Science will be searched, from their inception onwards, to identify relevant articles [14, 15]. Grey literature will be identified through search in Google Scholar and other relevant documents such as dissertation databases and government reports. The combination of keywords to use in the search strategy will be “cécité des rivières” OR “onchocercose” OR “onchocerciasis” OR “river blindness” OR “onchocerca volvulus” OR “simulium” OR “simulie” OR “blackflies” AND “cameroun” OR “cameroon”. A draft search strategy in PubMed/MEDLINE is available in Additional file 2. The reference lists of examined full-text papers will be scrutinized for additional relevant articles. Authors of primary publications or aggregated data and stakeholders involved in research and/or control of onchocerciasis in Cameroon (National Onchocerciasis Control Programme (NOCP) and NGDOs) will be contacted to request for unpublished data and/or resources (reports, datasets) relevant for this study.

### Study selection

The web-based software platform Covidence (www.covidence.org) will be used to manage the records obtained with the search strategy (citation screening, full-text review, risk of bias assessment, extraction of study characteristics and outcomes, export of data and references … ). In addition, two investigators will independently screen the studies identified by the searches following a two-step procedure. The first step will consist in the screening of titles and abstracts for relevance and exclusion of published literature that does not fulfil the eligibility criteria. The second step will consist in the review of full texts of potentially relevant and completely relevant literature identified during first screening step to assess their eligibility. In case of disagreements, a third investigator will be involved as an adjudicator, either by consensus or by discussion.

### Data extraction and management

Data will be extracted onto an Excel spreadsheet containing relevant information for the objective of the systematic review (see data items below). This will be performed by two independent reviewers, and any disagreements will be resolved by a third reviewer. When data will not be available (or unclear) in the manuscripts, the authors of the studies may be contacted for clarification.

#### Data curation

Prior to data extraction per se, a reference manager software (EndNote/Zotero) will be used to manage the retrieval of literature and to screen for and exclude duplicates. This will be done first automatically using the “find duplicate” or “de-duplication” function under EndNote or Zotero, respectively, by comparing the title or various combinations of the author(s), year, secondary title, volume, issue and page numbers. In the second instance, the records of suspected duplicates will be visually inspected.

#### Data items

After curation of the database, the full texts of the studies adhering to all the above eligibility criteria will be read, and a Microsoft Excel spreadsheet will be used for data extraction. The following items will be extracted: (1) publication title, (2) year of publication, (3) year(s) of data collection, (4) authors’ names, (5) location of the study (region, health district, community), (6) geographical coordinates (latitude and longitude) and altitude, (7) type of environment (forest, savannah, forest-savannah mosaic, type of vegetation), (8) climate data (temperature, precipitation, humidity), (9) river basin and proximity of study location to rivers/vector breeding sites, (10) intervention information (MDA, drug used for MDA, number of treatment rounds, duration of treatment, frequency, time interval between treatment and parasitological assessment, geographic coverage, minimum and maximum therapeutic coverage, treatment adherence, vector control, method for vector control), (11) human sample size, (12) age (and sex) of enrollees (maximal, minimal and median age (for males and females if available)), (13) assessment tool (skin snips for assessment of microfilaridermia with details on type of punch, incubation medium, incubation time, enumeration of microfilariae, weight of snips; nodule palpation; serological assay), (14) nodule data (prevalence, minimum, maximum and mean number of nodules), (15) skin snip data (microfilarial prevalence, minimum, maximum and mean number of microfilariae per skin snip or per milligrams of skin, community microfilarial load (CMFL)), (16) serological data (age groups tested, antigen used, seroprevalence), (17) blackfly data (sample size; biting rate; diagnostic tool for detection of infection in blackflies; number of flies with L1, L2 and L3; number of L3 per fly; minimum, maximum and mean number of L3 in the head/body), (18) vector identification and method (morphotaxonomy, cytotaxonomy, other), vector species composition, vector bionomics, as well as (19) co-endemicity with loiasis.

### Outcomes and prioritization

The primary outcomes will be (1) the prevalence and intensity of *Onchocerca volvulus* infection in humans (measured by microfilaridermia and palpable nodule data), (2) transmission intensity [measured by entomological indices such as vector biting rates (the number of flies/person/year), infective biting rates (the number of infective flies/person/year), and transmission potentials (the number of infective larvae/person/year)] and (3) impact of interventions on prevalence, intensity and transmission of onchocerciasis (measured by proportional changes in microfilarial prevalence and density between different data collection timepoints). The secondary outcomes will be environmental and socio-demographic factors supporting the primary outcomes (evaluated through meta-regression in the first instance and subsequently through spatial statistics techniques as described below).

#### Data analysis

Data will be recorded as point prevalence and/or mean intensity of infection (the type of mean, arithmetic or geometric, will also be recorded); uncertainty regarding sampling sizes will be estimated by calculating 95% confidence intervals for prevalence [16]. Since the distribution of the number of microfilariae per skin snip (or per milligrams of skin) and the number of palpable nodules per person is typically overdispersed among hosts, the standard deviation (SD) for intensity of infection will not be appropriate. However, when individual data are available, the distribution of these data will be ascertained and appropriate measures of variance will be calculated. For harmonization purposes, nodule prevalence will be converted into microfilarial prevalence [17, 18], albeit retaining information to allow identification of directly measured or converted microfilarial prevalence estimates. Prevalence and intensity of infection estimates will be stratified according to gender, age, geographical location and year of publication. Chi-square and the non-parametric Mann-Whitney and Kruskal-Wallis tests will be used to compare, between different data collection time points/periods, the prevalence and intensity of *O. volvulus* infection, respectively. Random-effects meta-analysis will be conducted. The *I*^2^ statistic will be used to quantify heterogeneity, and tau-squared (*τ*^2^) will be used to indicate the extent of between-study variance [19]. Meta-regression will be used to identify covariates influencing the estimates with the aim of identifying models that best predict the variability of effect sizes (i.e. that maximise maximum likelihood, minimise Akaike Information Criterion and explain the highest percentage of the dependent variable variance) [20, 21]. Publication bias and sensitivity analyses will also be conducted. (Publication bias can be assessed using funnel plots and Egger’s regression asymmetry test [22, 23]. A trim-and-fill technique to identify and correct the asymmetry in funnel plots can also be applied [24].)

Data on prevalence and intensity of infection will be used to draw thematic maps using a geographical information system (GIS) software (ArcGIS, version 10.2, ESRI Inc.), according to interventions and environmental characteristics. Indeed, maps of interventions against onchocerciasis as well as maps of different environmental parameters (climate, vegetation, hydrography, soil … ) will be superimposed on point prevalence and intensity of infection maps to elucidate the impact of these factors on onchocerciasis trends in Cameroon. Kriging and/or model-based geostatistical methods will then be used to obtain smooth prevalence maps throughout the country [25, 26].

### Assessment of risk of bias

The risk of bias of primary observational studies will be evaluated using a methodological quality critical appraisal checklist proposed in the Joanna Briggs Institute (JBI) systematic review methods manual [27].

There might be a bias in the conversion of prevalence of nodules to prevalence of microfilaridermia data since the statistical relationship between these two types of data is subject to a substantial amount of uncertainty. In addition, studies that will not present data disaggregated by community/village will not be included, likely leading to a selection bias.

### Confidence in cumulative evidence

The quality of the evidence will be judged using the Grading of Recommendations Assessment, Development and Evaluation (GRADE) approach [28]. Evidence quality assessment will be performed for each outcome. The grades of evidence will be defined into four categories and adjudicated as “high” (further research is unlikely to change our confidence in the estimate of effect), “moderate” (further research is likely to have an important impact on our confidence in the estimate of effect and may change the estimate), “low” (further research is very likely to have an important impact on our confidence in the estimate of effect and is likely to change the estimate) and “very low” (any estimate of effect is very uncertain) [28]. The confidence in evidence will be discussed among authors, and a narrative synthesis of the results will be provided as some degree of heterogeneity will be expected.

## Discussion

Onchocerciasis remains a public health problem, particularly in Africa (including Cameroon) where 99% of the infected population live. Despite control efforts through MDA with ivermectin, vector control and recent intervention trials of anti-*Wolbachia* doxycycline therapy, the infection still persists, with unexpected high prevalence in certain areas [6]. Indeed, it has been suggested, using epidemiological models, that onchocerciasis may not be eliminated before 2030 if interventions rely solely on ivermectin MDA [7–9, 29]. This systematic review will evaluate onchocerciasis epidemiological trends in association with environmental characteristics and control interventions in Cameroon. The outcomes of this systematic review will directly inform both programme managers and policymakers of available data and data needs. This will change the approach towards elimination of onchocerciasis in Cameroon by highlighting and characterising the various epidemiological scenarios that must be considered, the impact of current interventions and the need for tailored ATS. Based on a better understanding of the factors underlying spatiotemporal trends in onchocerciasis prevalence, intensity and transmission at ecologically appropriate scales (foci, river basins, community clusters), suitable combinations of alternative and complementary intervention strategies [11] will be defined to help accelerate onchocerciasis elimination wherever it is still endemic.

The main limitations of this systematic review will be (1) the access to unpublished data, or to more detailed information when data will be too aggregated, and (2) the discrepancies between collection and reporting of information by different authors. To overcome this situation, study authors of primary publications and/or collaborators will be contacted to collect additional information.

## Amendments to protocol

After approval and publication of the protocol, any amendments, if necessary, will be registered with PROSPERO and documented in the final publication. The date of each amendment as well as a description and rationale of each change made when publishing the review will be provided.

## Dissemination

This systematic review will be conducted as part of a PhD thesis. The results of this systematic review will be published in international peer-reviewed journals and disseminated to the research community, programme managers, policymakers and stakeholders through presentations at scientific and other meetings, via social media and mass media.

## Data Availability

The datasets used and/or analysed during the current study are available from the corresponding author on reasonable request.

## Abbreviations

ATS: Alternative treatment strategy
APOC: African Programme for Onchocerciasis Control
CMFL: Community microfilarial load
EMBASE: Excerpta Medica dataBASE
GRADE: Grading of Recommendations Assessment, Development, and Evaluation
MDA: Mass drug administration
NOCP: National Onchocerciasis Control Programme
NCEOLF: National Committee for Elimination of Onchocerciasis and Lymphatic Filariasis
OCP: Onchocerciasis Control Programme in West Africa
PUBMED: National Library of Medicine
